# Hospital avoidance: an integrated community system to reduce acute hospital demand

**DOI:** 10.1017/S1463423619000756

**Published:** 2019-10-29

**Authors:** Graham McGeoch, Brett Shand, Carolyn Gullery, Greg Hamilton, Matthew Reid

**Affiliations:** 1 Clinical Leader, Canterbury Initiative, Canterbury District Health Board, Christchurch, New Zealand; 2 Clinical Writer and Analyst, Canterbury Initiative, Canterbury District Health Board, Christchurch, New Zealand; 3 General Manager, Planning and Funding Department and Decision Support, Canterbury District Health Board, Christchurch, New Zealand; 4 Team Leader, Intelligence and Transformation, Planning and Funding Department, Canterbury District Health Board, Christchurch, New Zealand; 5 Public Health Physician, Planning and Funding Department, Canterbury District Health Board, Christchurch, New Zealand

**Keywords:** acute demand, ambulatory care, emergency services, hospital, growth and development, home care services, primary health care

## Abstract

**Background:**

Growth in emergency department (ED) attendance and acute medical admissions has been managed to very low rates for 18 years in Canterbury, New Zealand, using a combination of community and hospital avoidance strategies. This paper describes the specific strategies that supported management of acutely unwell patients in the community as part of a programme to integrate health services.

**Intervention:**

Community-based acute care was established by a culture of close collaboration and trust between all sectors of the health system, with general practice closely involved in the design and management of the services, and support provided by hospital specialists, coordination and diagnostic units, and competent informatics. Introduction of the community-based services was aided by a clinical guidance website and an education programme for general practice teams and allied health professionals.

**Outcomes:**

Attendance at EDs and acute medical admission rates have been held at low growth and, in some cases, shorter lengths of hospital stay. This trend was especially evident in elderly patients and those with ambulatory care sensitive or chronic disorders.

**Conclusions:**

A system of community-based care and education has resulted in sustained gains for the Canterbury health system and freed-up hospital resources. This outcome has engendered a sense of empowerment for general practice teams and their patients.

## Background

Increases in attendance at Emergency Departments (EDs) and acute admissions impact adversely on the quality of care, delay elective surgical procedures, and expose patients unnecessarily to the risks of hospital admission (New Zealand Health Technology Assessment, 1998; Purdy and Huntley, 2013). This paper provides an overview of the development and impact of an Acute Demand Management Service (ADMS) in the Canterbury region of New Zealand. This system was introduced with the purpose of reducing acute ED attendances, paediatric admissions, and adult medical admissions to hospitals by increasing access to community-based services provided by extended general practice teams. The foundation required to develop these services was established by health care reforms that occurred in New Zealand in the mid 1990s, one of which was the formation of independent practitioner associations (IPAs) by groups of general practices. The aim of these IPAs was to coordinate primary care services and provide infrastructure support, while leaving individual general practices to remain as independent businesses.

Prior to 2000, health care in New Zealand was provided by fully funded public hospitals, private hospitals, and a partially subsidised primary care system, which charged patient variable fees for services over and above the subsidies received from the government. Access to primary care services for patients could, therefore, be expensive, and often secondary care services were used as a ‘free to the patient’ substitute. For acute care, general practice teams tended to only provide basic care with many admissions to public hospitals not involving a prior consultation with a general practitioner (GP) and hospital-based services being the social norm for acutely unwell patients. This led inevitably to unsustainable growth in acute admissions to public hospitals.

In 2001, as part of legislative changes, 21 geographically defined District Health Boards (DHBs) were established across New Zealand, with each DHB receiving population-based funding from central government to fund both secondary and primary health care (New Zealand Ministry of Health, 2001; Cumming and Mays, 2002). Distribution of the funds for primary care was administered, in turn, by Primary Health Organisations (PHOs), with funding adjusted according to the demographic characteristics of the patients enrolled with general practices within the PHO. Initially, five PHOs were established in Canterbury, which have been consolidated to three, the largest of which comprised the previously formed IPA and was called Pegasus Health (https://www.pegasus.health.nz/). Since being established, Pegasus Health had worked to organise, integrate, and educate general practice with the belief that they could play a greater role in the local health system (Kirk *et al*., 2002; Ali and Rasmussen, 2004). It was recognised that the range of skills of general practice teams were being under-utilised and that the issue of acute medical admission growth could be alleviated, in part, from a primary care base. Drawing together these themes, the ADMS was developed to increase the availability of care for acutely unwell people in their own homes (Timmins and Ham, 2013; Charles, 2013; Gullery and Hamilton, 2015; Pegasus Health, 2017). The emphasis was on overcoming the logistical and financial barriers to accessing diagnostic, treatment, and support services in the community. General practice doctors and nurses were therefore trained, re-educated, and supported to provide and coordinate increased acute care. A contract was signed between Pegasus Health and health funders in which Pegasus Health held the budget and shared the financial risk for growth in acute medical admissions with Christchurch Hospital. Pegasus had the funds to participate in the ADMS because they had retained funds from budget holding for laboratory and pharmaceutical services undertaken in the 1990s (Pegasus Health, 2017). The ADMS was later extended to include general practices aligned with other PHOs in Canterbury.

**Figure 1.**
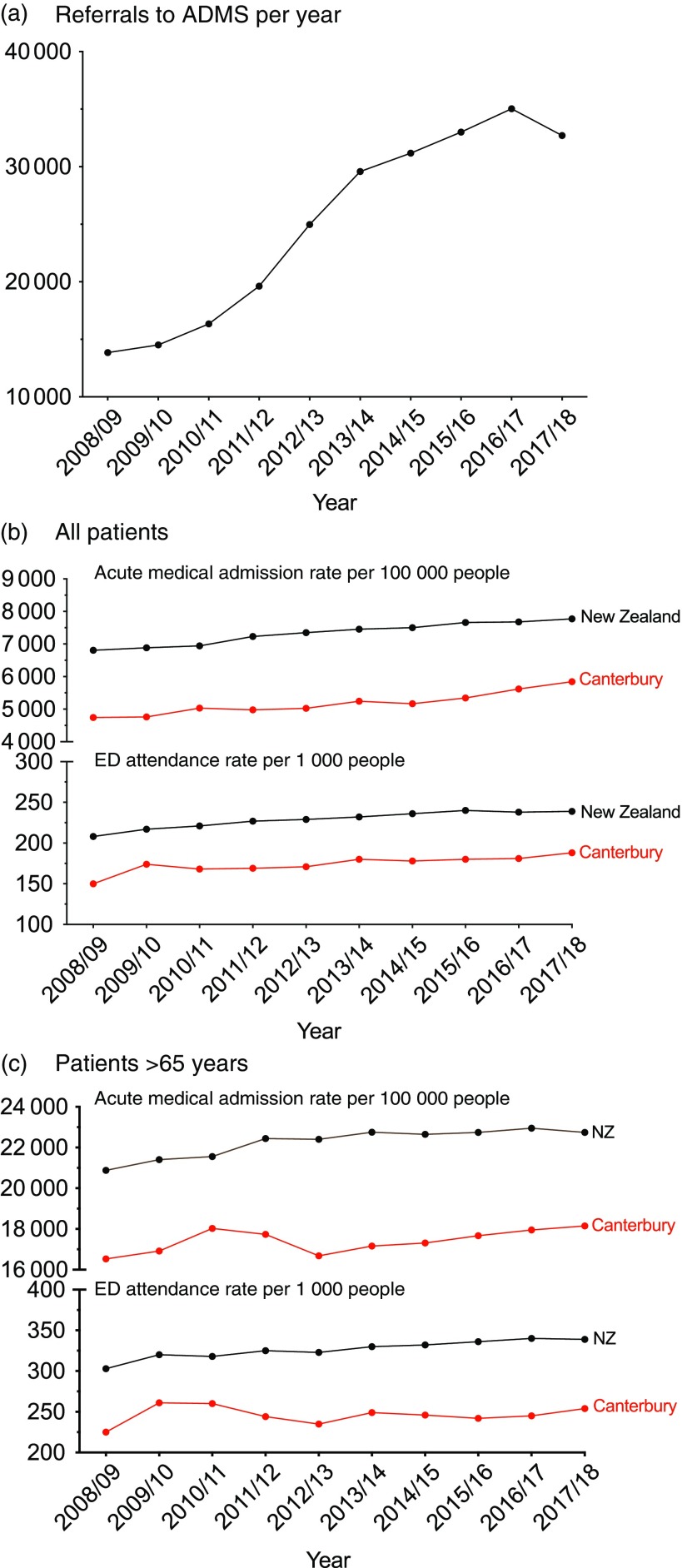
Graphs showing the impact of the acute demand management service between 2008 and 2018 on (a) number of referrals each year to the service, and rates of attendance to the Emergency Department and acute medical admissions for (b) all patients and (c) patients older than 65 years.

Development and expansion of the ADMS has resulted in a progressive shift towards services in the community. For example, in the last 10 years, there has been a 2.5-fold increase in referrals to the ADMS, with approximately 34 000 patients in the funded population of 558 830 currently treated every year (Figure 1a). Data collected in 2015 showed that Māori and Pacific peoples had higher age-standardised utilisation rates of the ADMS (World Health Organisation, 2001) than European and Others. People of Asian ethnicities had lower utilisation rates.

Compared to the mean ED attendance rate in New Zealand, Canterbury has maintained a lower level of ED presentations and acute medical admission rates over this period (Figure 1b). These improvements have been especially apparent among elderly people, with the combination of the ADMS and the Community Rehabilitation Enablement and Support Team (CREST) maintaining lower rates of ED attendance and acute admissions in an aging society (Figure 1c). While this paper describes improvements in acute services, Canterbury has also undertaken a much broader integration programme covering longitudinal care, which is different from the rest of New Zealand. The contribution of ADMS to the observed changes can, therefore, not be considered in isolation from the contribution of high quality, readily accessible routine care.

The potential of programmes such as the ADMS was reviewed in a recent paper by Toop (2017) who emphasised the need for such systems ‘to be trialed and evaluated at scale’. In this context, the following sections describe the impact of the ADMS and discuss the principles and strategies used to develop the programme.

## Design and introduction of the ADMS

The ADMS was designed around the results of a pilot study in a group of local general practices that assessed the range of services GPs felt would be useful to manage patients in the community who previously would have been referred to the hospital. The pilot study identified variability in referral patterns and admission behaviour for acute care. This variability was potentially modifiable by ready access to community-based diagnostic and treatment services combined with training and funding in specific areas. Supporting evidence from reports of international healthcare organisations (Jones and Smith, 2000) led to several projects to vertically integrate health services from a primary care base. The projects aimed to provide flexibility, rather than protocols, that would enable general practice teams to access the services the doctor and the patient felt would be most useful for that patient. This involved a level of devolved financial trust from Pegasus Health, which initially held the risk for the budget, to the member general practices. This model of care differed from earlier integrated home-based health care programmes (Brazil *et al*., 1998; Richards *et al*., 1998; Shepperd *et al*., 1998) because it was developed and managed by primary care rather than acting as an outreach programme from hospital-based services.

**Figure 2.**
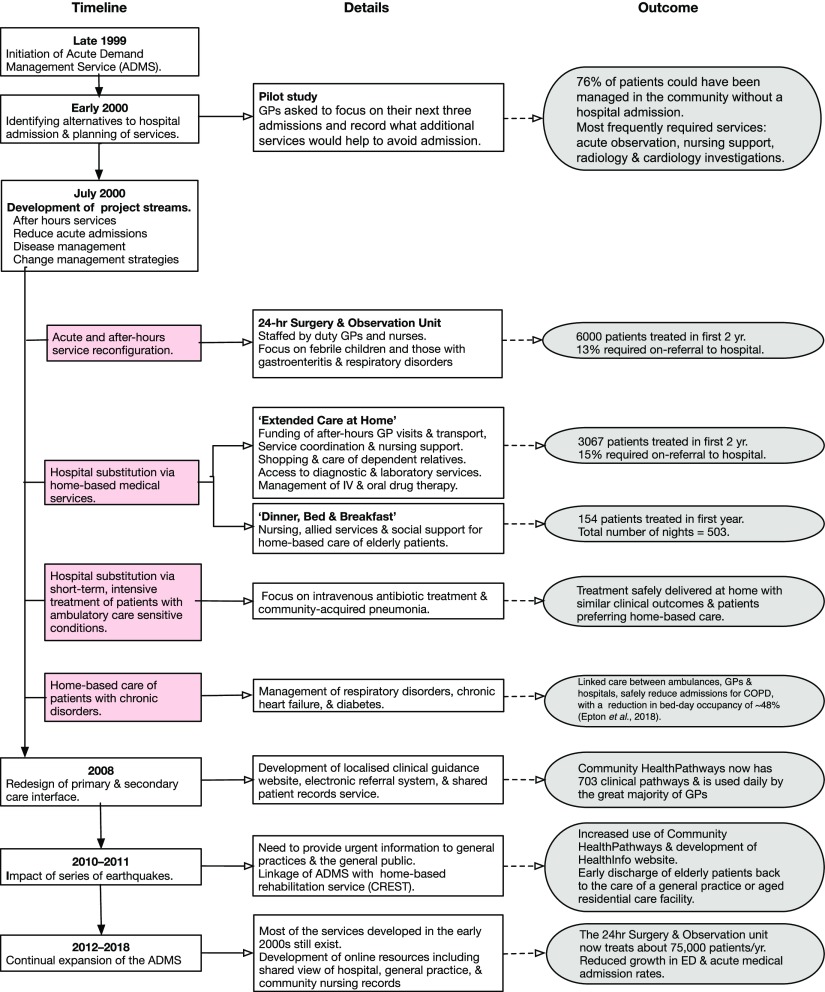
Flow diagram showing the timeline of the development of the acute demand management service.

The development and growth of the ADMS over the next 18 years is shown in Figure 2. The philosophy of the service design was to provide whatever it took to safely look after patients at home. General practice teams had flexible funding to a maximum value available to look after patients they would otherwise have sent to hospital. This removed the financial barriers to care in the community, with the services used iteratively to inform further service development. The initiatives included services that were not disease specific and applied to all age groups:

1. Care provided in a 24-h family-friendly Observation Unit by experienced paediatric nurses or observation in the patient’s general practice. GPs have to make quick decisions about the need to admit patients. Particularly for young children, watching patients for 1–2 h and providing some basic care and parental instruction reassure the patient, family, and doctor that care can be provided at home or confirm the need for hospital admission.
2. Funding of usual GP and nurse home visits and general practice attendances to continue to look after patients during their acute illness. Without the ADMS, these repeat visits were at a cost to the patients.
3. Alternative follow-up services provided by specific doctors and nurses contracted to Pegasus Health. This meant GPs could put a patient ‘On Acute Demand’ and still go off duty, knowing that good home care would continue.
4. Flexible, automatic, minimal or no form-filling, and no questions asked regarding acceptance of requests.
5. Access to extra nursing assessment and care by well-equipped, mobile nurses.
6. Access to rapid diagnostics including radiology, ultrasound, ECG, and blood tests. The test results were made available to the teams as quickly as in the hospital, while community-based radiology was carried out either immediately or on the same day (Holland *et al*., 2017).
7. Specialist phone support. A co-ordination centre staffed by registered nurses provided a 24-h telephone triage system and arranged access to services as required in response to an urgent phone call from a general practice.
8. Rapid access to geriatric rehabilitation services (CREST) was a later addition in 2011.
9. Disease-specific acute services backed by improved routine care of patients with long-term disorders.

For patients suitable for community-based acute care, general practice teams were provided with two options. The first, ‘Extended Care @ Home’, combined the concepts of ‘Hospital at Home’ (Wilson and Parker, 1999; Shepperd and Lliffe, 2005) and the ‘Roving Ward Round’ (Sibbald *et al*., 2007), but was implemented from a general practice not hospital viewpoint. This service allowed a GP to authorise up to three days of home-based nursing and logistical support with no conditions attached. The second option, ‘Dinner Bed and Breakfast’, was available for elderly patients requiring a lower level of support where accommodation in aged residential care was arranged (Hanger *et al*., 2005). This care was directed towards patients who were usually independent and focussed on convalescence for patient recovery, rather than active rehabilitation, that could only be arranged by further referral. This limitation was addressed in 2011 by the development of the CREST programme which recognised rehabilitation was better provided at home and avoided patients becoming institutionalised in an aged residential care facility.

Better out-of-hospital care, by itself, may be insufficient to offset the demand for acute hospital services. The ADMS was, therefore, extended to include disease-specific hospital substitution services. These focussed on short-term intensive treatment for patients with ambulatory care sensitive conditions that included intravenous antibiotic therapy for cellulitis and pneumonia, diagnosis of deep vein thrombosis, management of heart failure and exacerbations of chronic respiratory diseases (McGeoch *et al*., 2006), and short-term care for elderly people and acutely unwell children in the community. Another option was to arrange home-based management of patients with chronic disorders with high morbidity, as it was known that this patient group formed a major proportion of acute demand (Rothman and Wagner, 2003; Verhaegh *et al*., 2014; Chapman *et al*., 2018). Each disease-specific programme consisted of agreed clinical guidelines supported by coordination and increased availability of services. Formal review in 2003 (Law and Economic Consulting Group, 2003) showed that these two services resulted in fewer ED presentations and hospital bed days related to the conditions, particularly respiratory. Randomised controlled studies of patients treated for either cellulitis (Corwin *et al*., 2005) or community-acquired pneumonia (Richards *et al*., 2005) showed no difference in clinical outcomes or cost of community care compared to hospital-based treatment and that patients preferred to stay out of hospital when possible. While the average claim in 2003 was quite low at approximately NZD 200 (equivalent to approximately 120 USD or 70 British Pounds at 2003 exchange rates), these services promoted a high level of teamwork between primary and secondary care clinicians, nursing staff, and allied health professionals to achieve successful clinical outcomes.

## Governance and funding of the ADMS

Since 2011, an alliance contracting system has been used to fund the various ADMS services. This system has evolved from models of collaboration used in the construction industry (Ariño and Reuer, 2004; MacDonald *et al*., 2012) and has become a broadly accepted procurement and delivery method that has been used in complex projects. Under such contracts the funder, a public sector agency, works collaboratively with private sector subcontractors in good faith and integrity to make consensus decisions on all key issues for project delivery. The approach capitalises on existing relationships between funders and contractors to remove organisational barriers and promote effective integration (Timmins and Ham, 2013). In this way, clinical leadership is encouraged with groups of health professionals working together to achieve shared goals and agree on the best use of health care resources for both the patients and the overall health system (Miller, 2009; Burns and Briggs, 2018). The contracts require an act of faith both from funders to pay for the services and from general practice teams to ensure the extra resources are used responsibly and effectively. Overview and governance of the ADMS is carried out jointly by Pegasus Health and the Canterbury Clinical Network (http://ccn.health.nz/), an alliance of healthcare providers from across the entire Canterbury health system. Claims for services are made via an e-portal managed by Pegasus Health with fee for service payments made for an ‘episode of care’ (Pegasus Health, 2017). Indicative fee guidance is provided, and the small number of invoices felt to be excessive is managed through clinician-to-clinician conversations.

## Early impact of the ADMS

**Figure 3.**
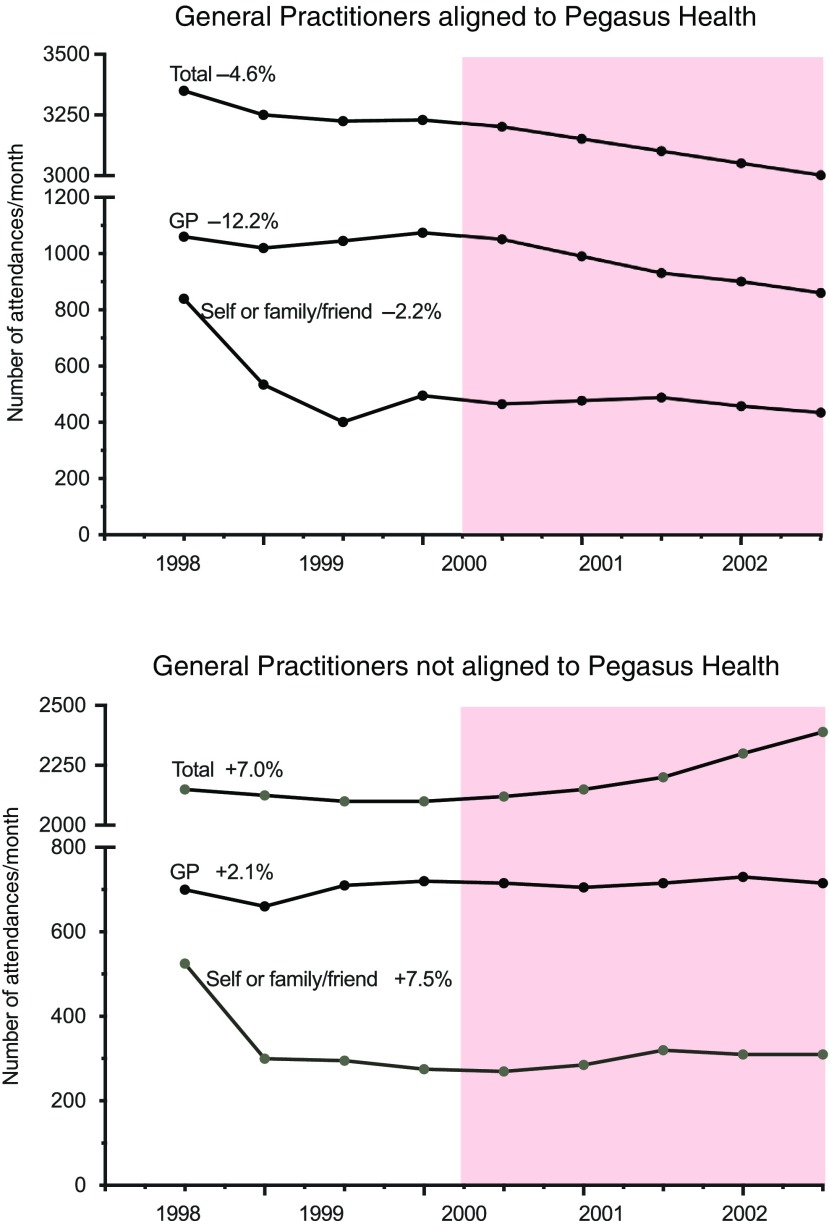
Comparison of attendance at the Emergency Department between 1998 and 2002 of patients aligned to general practices participating in the acute demand management service and those aligned to non-participating practices. The shaded area shows the period following introduction of the ADMS.

In the first four years, the ADMS was available only to general practices within the Pegasus Health, before being extended across Canterbury in 2004 when funding of the service was transferred to the recently formed Canterbury DHB. These early years provided an opportunity to compare changes in utilisation of health services between Pegasus Health and non-Pegasus Health members following introduction of the ADMS (Law and Economic Consulting Group, 2003). At the start of the programme, patients under the care of Pegasus Health-aligned GPs accounted for approximately 60% of acute ED attendances. As shown in Figure 3, a reduction of 4.6% in ED attendance occurred for patients managed by Pegasus Health-aligned general practices in the first two years of the ADMS compared with an increase of 7% for patients cared for by non-Pegasus Health-aligned practices. This reduction for Pegasus Health-aligned patients occurred as a result of a lower number of GP referrals and fewer people coming to the ED with their family without having previously been seen by their GP. An increase in the acuity of the referrals was apparent with the proportion of GP referrals that become acute admissions increasing by 15% for Pegasus Health-aligned GPs compared with a 5% decrease for non-Pegasus Health GPs.

## Hospital initiatives to manage acute admissions

The ADMS was complemented by a number of changes in patient management in the ED introduced to make the best use of specialised hospital services and to strengthen community-based services (Ardagh, 2015; Gullery and Hamilton, 2015). This involved attempting to alter the default option of care for people presenting to the ED, rather than the embedded practice where management of many cases was unintentionally skewed towards admission irrespective of the severity of the condition (Elley *et al*., 2007). Where appropriate, patients were deflected from ED to primary care for non-urgent and on-going management of their illness. For patients who were admitted, the strategy was to facilitate structured discharge to primary care for patients who could be cared for in the community.

## Improving the design of the primary and secondary care interface

Better pre- and post-referral management of patients by general practices was recognised as a key factor for maximizing the effectiveness of the ADMS. A project known as the Canterbury Initiative (https://www.cdhb.health.nz/about-us/key-projects-and-initiatives/canterbury-initiative/) evolved, with primary and secondary care clinicians and hospital managers forming work groups to construct clinical pathways for several commonly referred conditions. These pathways were centred on international best practice guidelines to ensure optimal pre-referral management and agreed access criteria for secondary care and structured so that the information was easy to view during a patient consultation. To disseminate this information, a password-protected website was constructed and went live in October 2008 under the name, Community HealthPathways (https://www.streamliners.co.nz/HealthPathways.aspx).

This website is a unique combination of clinical guidance, information on local health system processes, and directory services. Currently, there are over 700 clinical pathways on the website with information provided on all common ambulatory care sensitive conditions (McGeoch *et al*., 2015a; 2015b). Community HealthPathways was subsequently linked with an electronic request management system that allowed general practices to send structured referral information to a central database and then to community and hospital services (http://www.cdhb.health.nz/Patients-Visitors/Pages/Referrals.aspx).

## Impact of the Christchurch earthquakes

A series of earthquakes occurred in Canterbury between 2010 and 2012, with a major quake in early 2011 causing 185 deaths and widespread damage to healthcare infrastructure, particularly Christchurch Hospital (McIntosh *et al*., 2012; Schluter *et al*., 2016). This disaster focussed urgent attention on the need for safe community options for acute care and resulted in accelerated development of several services in the ADMS. This was particularly apparent for Community HealthPathways which was used to disseminate urgent information to general practice teams, pharmacies, and community nursing services and prompted development of a website called HealthInfo (http://www.healthinfo.org.nz) to provide urgent information to the general public. Over time HealthInfo has been extended to contain similar information to that supporting the pathways on Community HealthPathways, written in an easy-to-understand style. Patients are encouraged to seek general practice care first and not to attend the ED except for emergencies, with self-care in the community for complex patients guided by shared management plans prepared by their GP.

The effects of the earthquakes on the ADMS were especially evident for people older than 65 years who accounted for approximately 70% of acute hospital bed days. In 2011, CREST was established to provide clinical assessment and home-based rehabilitation of elderly patients for up to six weeks and individualised care plans for long-term use in the patient’s home. This resulted in general practice teams having improved access to rehabilitation services and domiciliary support for elderly patients. The linkage between ADMS and CREST was subsequently extended so that general practice teams could refer patients directly to CREST, with the service now delivering approximately 1900 episodes of care annually and many patients avoiding hospital admission.

## Learnings

**Figure 4.**
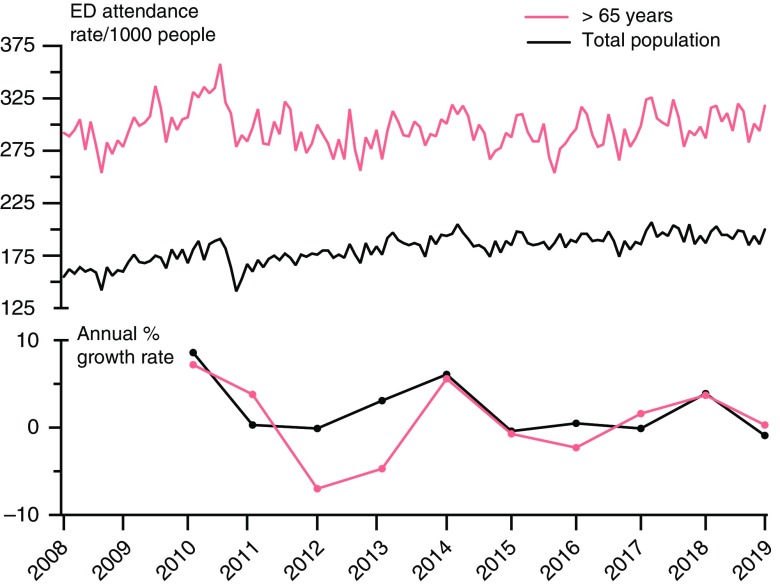
Number and growth rate of attendances to the Emergency Department between 2008 and 2019 (six months data only) for the total population and people older than 65 years.

There is only limited evidence on the effectiveness of coordinated system-wide and home-based services to manage acute and urgent care (Purdy and Huntley, 2013; Whittaker *et al*., 2016; Toop, 2017). Questions remain as to whether the gains achieved by the services are sufficient to reach national targets for reductions in hospital acute admissions (Damery *et al*., 2016). Both the national data and local comparative data described in this paper indicate that acute demand for hospital services can be managed primarily by general practice teams linked to community-based services and supported by secondary care clinicians. Nationally, Canterbury has maintained lower rates of ED attendance and acute medical admission compared with other regions in New Zealand, with sustained lower rates in hospital bed days and case weights for conditions treated in the community (Love, 2013). Early local data confirmed that these improvements were only observed in patients of general practice teams participating in the ADMS programme. In addition to maintaining a lower rate of ED attendances, there has also been a recent slowing in the growth of these attendances, from approximately 3% between 2011 and 2017 to 0.5% currently (Figure 4). This trend may be due, in part, to the diminishing impact on acute health services of the large workforce required to repair earthquake damage and the successful collaboration between the ADMS and CREST for community-based care of elderly patients who make-up a large proportion of ED admissions.

Further reductions in acute demand were achieved by improving standards of care in general practice, promoting continuity of care for patients with ambulatory care sensitive conditions, and improving care transition from hospital to home for those with common chronic disorders. This required hospital staff being aware of the community services available so that only high-acuity patients were admitted and for those admitted having the option to arrange prompt discharge to home-based care with a management plan in place. This strategy is consistent with the findings of other studies that reported patients preferred to have their care in the community when possible (Montgomery-Taylor *et al*., 2016; Barker *et al*., 2017).

For there to be a sustained reduction in the number of ED attendances, it is necessary to normalise self-management where appropriate and reorient the population to seek assistance in primary care, and only attend the ED for emergencies. To ensure equity of outcomes from the ADMS, it is especially important that access to it through general practice and information about it is appropriate for people who experience greater risk and prevalence of disease such as Māori and Pacific peoples and those in lower socioeconomic groupings. This change in behaviour is dependent on maintaining effective, adaptable, and affordable services in general practice and providing greater access to community-based services to manage acute care, such as the 24-h Observation Unit described earlier in this paper.

Despite a trend worldwide of general practice withdrawing from urgent and acute care (Raymont *et al*., 2005; Pitts *et al*., 2010; Chauhan *et al*., 2012), why have general practice teams in Canterbury been willing to participate in care that is often outside working hours, disruptive to normal general practice appointments, and that may require home visits? A systematic review of health integration models concluded that the 10 elements listed in Table 1 were essential for successful and sustained integration (Nicholson *et al*., 2013). The ADMS appears to encompass all these elements, the most important of which were the shift in culture and improvement in working relationships between primary and secondary care clinicians and the fact that the ADMS was designed primarily by general practice. These changes necessitated a departure from previous referral behavior, while relying on the clinical judgement of general practice teams in the absence of control by a detailed process or rigid guidelines. With the support of rapid diagnostic services, the intention was that GPs would gain confidence that they were doing the right thing and would be supported by their hospital colleagues, irrespective of whether hospital admission was eventually required. In cases where a GP did not have the knowledge or time to deliver services for patients or wished to hand over care at night or weekends, the ADMS provided flexibility and centralised alternatives for acute care outside the hospital. As a consequence, general practice teams gradually became socialised that urgent care in the community was an expected part of their daily routine without necessarily impacting on their practice time and lifestyle. As with all behavior in general practice, there remains a wide variation in the use of ADMS by different GPs.

**Table 1.** Elements for successful and sustained integration

Element	Relevance to the ADMS
Joint planning	Promotes a community focus and primary care organisational autonomy.
Integrated information communication technology	Provision of online clinical guidance, referral criteria, and directory of local health services and resources. Shared electronic health records with summary information to clinical teams (HealthOne).
Change management	Managed locally, committed resources, Alliance contracting, and executive and clinical leadership.
Shared clinical priorities	The ADMS involves multi-disciplinary network with pathways across the continuum of care.
Incentives	General practice teams and others were fairly funded for their services to reduce barriers to participation. There were deliberately no financial incentives for participation.
Population focus	The ADMS focusses on a geographical population with the aim of caring for people in the community.
Using data as quality improvement tool.	Process and outcome data are shared to examine the impact and utilisation of services and to redesign these if necessary.
Continuing professional development and supporting the value of joint working.	Inter-professional learning opportunities provided by an education and skill workshop programme.
Patient – community engagement	Patient and community groups collaborate in the planning and ongoing development of the services.
Innovation	Adequate resources are available and innovative models of care are supported. Clinical guidance is provided, but the pathways are not prescribed.

ADMS = Acute Demand Management Service.

To make management of acute demand as easy as possible for the general practice teams, the ADMS was based on an ethos of high trust with minimum bureaucracy, with only essential information being required to initiate any service in the programme. Additional information for claiming or monitoring of the patient was collected later, with claims up to NZ$500 (equivalent to approximately 300 USD or 175 British Pounds at 2003 exchange rates) paid without question, and higher claims subjected to increased levels of scrutiny, but only retrospectively. In this way money did not get in the way of providing urgent care, enabling a high trust environment. This change proved to be a major shift for managers and finance teams who previously had wanted prior authorisation of small claims, a requirement which would not have worked in a general practice setting with multiple brief patient contacts.

Successful introduction of the ADMS would not have occurred without a structured education programme for general practice teams and allied health professionals that offered learning opportunities in several different formats. For certain conditions, the delivery of care was dependent on education and skill enhancement of the general practice teams. Acute care was included in the Pegasus Health education and skills workshops programme (Richards *et al*., 2003), while the 24-h Observation Unit proved to be an excellent training ground for GPs in a supportive environment with highly skilled nurses and medical officers. Increased use and recognition of the value of online clinical guidance, eReferral systems, and electronically shared clinical records were apparent throughout the development of the ADMS, especially following the damaging Canterbury earthquakes. To offset the tendency for the public to regard the ED as their first option for acute care, patients were encouraged to engage with community-based services, including nurse-led telephone triage linked to general practice, simplifying the message to the population. HealthInfo helped people decide where and when to seek acute care and engendered confidence that safe care was available without attending the hospital, reinforced by social marketing that promoted an orientation towards general practice.

The ADMS faced a number of conundrums and challenges throughout its development. The first was implementing the changes across a complex system and a large number of different organisations and professions. The decision to fully implement the ADMS at scale rather than using a piloting approach proved important in the early stages of the programme, as measurable benefits were observed within a short period of time. Canterbury has the advantage of a population that is large enough to sustain the capacity of the primary care workforce, while being small enough to be highly responsive and able to implement services quickly. Another challenge was a perception locally that the DHB structure favoured hospital care over primary health care. Internationally, doubts had been expressed concerning the resilience of schemes seeking to reduce hospitalisation to be able to provide the necessary ongoing support to general practice teams, which commonly perceived the programmes are of limited value, time consuming, and resulted in financial disadvantages (Health Canada, 2002; New Zealand Ministry of Health, 2005). However, the design of the ADMS managed by primary care and not subject to externally imposed service specifications, however, changed the perception of local general practice teams. Over time, hospital clinicians have learned to trust care provided in the community and have provided increasing support to general practice teams either personally or by web-based clinical guidance.

Another concern was that clinical audit of the ‘Dinner Bed and Breakfast’ service showed that approximately 15% of elderly patients receiving short-term care in aged residential care facilities became institutionalised (Hanger *et al*., 2005). A similar trend had been observed in other countries (Choi and Liu, 1998), and therefore, the service was stopped, with the ideal of short-term home-based care for acutely unwell older people not being achieved until the ADMS was linked to a rehabilitation team (CREST). It has also been recognised that home-based services could possibly reduce quality of care and increase overall service volume, leading to a loss in economy of scale (Blatchford and Capewell, 1997). However, this doubt was allayed by audits that showed hospital substitution care pathways for lower risk, ambulatory patients were both effective and safe (Corwin *et al*., 2005; *et al*., 2005; McGeoch *et al*., 2006).

There are some limitations to the data described here. Because every aspect of an integrated health system supports management of acute demand, it is important to recognise that each element interacts to contribute to the outcome. In a complex adaptive system, attribution of reduced hospital utilisation is also difficult as factors other than the ADMS may have interacted with the service and influenced the results. For example, other services such as the Falls programme (http://ccn.health.nz/Portals/18/Documents/IPANZ%20-%20Falls%20Powerpoint.pdf) have contributed to the lower ED attendance we observed. Christchurch also had two extended hours urgent care centres and one large open-all-hours clinic prior to the introduction of the ADMS that managed relatively high-acuity patients. All three clinics are now larger and more capable than 18 years ago. In addition, Canterbury has lower levels of socioeconomic deprivation than other parts of New Zealand (Stats NZ, 2013), which may contribute to the sustained lower level of ED attendances and acute admissions to hospital.

Variations of the ADMS have been implemented in other regions of New Zealand. All these programmes are less well-resourced than in Canterbury and tend to offer restricted packages of care for specific conditions such as cellulitis. The non-specific nature of the programme in Canterbury with high trust and low barriers to access is the key differences with these other programmes. The current cost per head on a population basis of the ADMS for the Canterbury population is NZD 11.90 (USD 7.70, British Pounds 6.00), with the costs in other DHBs estimated to be significantly less because of the more restricted range of community-based services that they provide. It is possible that this focus on community-based care in Canterbury will result in a decrease in both the ED and acute medical inpatient costs *per capita*.

The reasons why the rest of New Zealand has not adopted primary care acute demand are complex and may include politics, ideology, inertia, different levels of general practice engagement and cooperation, and the lack of published evidence of the benefit of these systems. In New Zealand, there is a debate on the extent to which the DHB structure is inherently structured to favour hospital care over primary health care, with individual DHB attitudes to primary care varying. Some DHBs have adopted a strong focus on their wholly owned hospital services rather than fostering closer working relationships with contracted primary care services, while others, such as Canterbury DHB, regard their relationship with primary care as essential to their overall purpose.

## Conclusions

By identifying patients suitable for community-based treatment and providing practical assistance and follow-up to ensure their acute health needs had been met, general practice teams with support have been able to increase capacity to meet demand for urgent hospital services. This has engendered a sense of empowerment for these teams, achieved at a comparatively low cost per case. The increased capability of general practice to provide acute care was achieved by adequate resourcing, an ethos of high trust and low bureaucracy, phone and virtual support from specialist services, and gains in skill and knowledge from a comprehensive education programme.

The ADMS was strengthened by hospital substitution strategies for patients with chronic disorders with high morbidity and linkage to community rehabilitation services for elderly patients. Changes in culture and work relationships were critical to moving towards the shared goals of the ADMS, with decision-making being clinician-driven within an alliance framework. The ADMS has both helped to establish and in turn benefited from a system focussed on the integrated service provision. The individual practical strategies described in this paper have only succeeded within this context.
